# Study on microbial community of “green-covering” *Tuqu* and the effect of fortified autochthonous *Monascus purpureus* on the flavor components of light-aroma-type *Baijiu*

**DOI:** 10.3389/fmicb.2022.973616

**Published:** 2022-08-18

**Authors:** Liping Zhu, Lanqi Li, Qiang Yang, Liang Chen, Lei Zhang, Gang Zhang, Bin Lin, Jie Tang, Zongjie Zhang, Shenxi Chen

**Affiliations:** ^1^Hubei Key Laboratory of Quality and Safety of Traditional Chinese Medicine and Health Food, Jing Brand Co. Ltd, Daye, China; ^2^Hubei Key Laboratory of Edible Wild Plants Conservation and Utilization, College of Life Sciences, Hubei Normal University, Huangshi, China

**Keywords:** light-aroma-type *Baijiu*, *Monascus*, “green-covering” *Tuqu*, red heart, high throughput sequencing, flavor components, correlation

## Abstract

“Green-covering” *Tuqu* (TQ), as one of *Xiaoqu*, is a special fermentative starter (also known as *Jiuqu* in Chinese) that originated in southern China and is characterized by a layer of green mold covering (*Aspergillus clavatus*) the surface and (sometimes) with a red heart. It plays a vital role in producing light-aroma-type *Baijiu* (LATB). However, to date, the microbiota that causes red heart of TQ remain largely unexplored, and it is still unclear how these microbiota influence on the quality of LATB. In this study, two types of TQ, one with a red heart (RH) and another with a non-red heart (NRH), were investigated by high throughput sequencing (HTS) and directional screening of culture-dependent methods. The obtained results revealed the differences in the microbial communities of different TQ and led to the isolation of two species of *Monascus*. Interestingly, the results of high performance liquid chromatography (HPLC) detection showed that citrinin was not detected, indicating that *Monascus* isolated from TQ was no safety risk, and the contents of gamma-aminobutyric acid in the fermented grains of RH were higher than that of NRH during the fermentation. Selecting the superior autochthonous *Monascus* (M1) isolated from the TQ to reinoculate into the TQ-making process, established a stable method for producing the experimental “red heart” *Tuqu* (ERH), which confirmed that the cause of “red heart” was the growth of *Monascus* strains. After the lab-scale production test, ERH increased ethyl ester production and reduced higher alcohols production. In addition, *Monascus* had an inhibitory effect on the growth of *Saccharomyces* and *Aspergillus*. This study provides the safe, health-beneficial, and superior fermentation strains and strategies for improving the quality of TQ and LATB.

## Introduction

**FIGURE 1:**
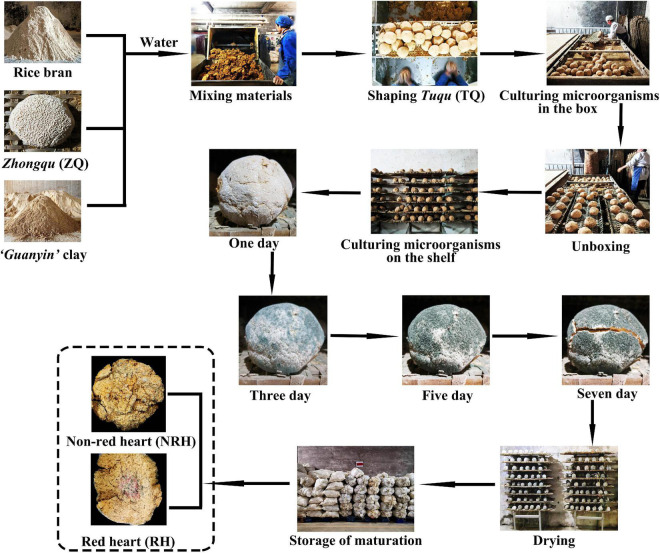
Schematic of the production process of “green-covering” *Tuqu*.

“Green-covering” *Tuqu* (TQ) is a complex microbial cocktail commonly used to brew light-aroma-type *Baijiu* (LATB) in southern China, which has a unique functional microbial composition and production process. The flavor of LATB brewed by TQ is mellow sweetness and refreshing aftertaste (Zheng and Han, 2016), and is popular with consumers (Jin et al., 2017; Fan et al., 2019). TQ is produced by the traditional process (Figure 1). The raw materials mainly consist of “*Guanyin*” clay, rice bran, and *Zhongqu* (ZQ). Among them, ZQ is a significant primitive seed culture for the production of TQ. After the mixing of raw materials, the TQ is shaped into a typical spherical or round cake shape, then, it is saccharified and cultivated in the box in one room for 1 day and on the shelf in another room for 7 days. It can be used in the workshop after storage for 2–3 months (Tian and Feng, 1999). The open manufacturing environment of TQ provides conditions for the enrichment of microorganisms. However, the traditional manual production process not only causes differences in the size and tightness of the TQ, but also leads to the differences in the organoleptic properties of TQ. Currently, the description of organoleptic properties is often used as an evaluation parameter to characterize the quality of the TQ. For example, the amount of “green coat” formed by *Aspergillus clavatus* growing on the surface of TQ is a crucial parameter for judging the quality of TQ. While *Aspergillus clavatus* affects the growth of other microorganisms when there is too much “green coat”, an excessively low concentration will lead to insufficient microbial diversity (Feng et al., 2004; Li et al., 2010). In addition, in the production process of TQ, the character of red color in the center of TQ (red heart) is frequently encountered. According to the practical production experiences, the TQ with red heart is often considered to be a reflection of high quality. In previous studies, a few yeasts, molds, and bacteria (Hu et al., 2021a,b; Tang et al., 2022), including *Monascus* were isolated from LATB. Then, Zhu et al. (2014) reported that three *Monascus ruber* were isolated from red-heart Langjiu *Daqu* by plate separation. However, there are still many unsolved problems derived from the lack of research on the red heart during the process of TQ-making, and it is unknown whether *Monascus* is the microorganism that causes the red heart of TQ.

*Monascus* is a medicine and food homologous microorganism, featuring the characteristics of lactic acid tolerance, ethanol tolerance, and hypoxia tolerance (Weusthuis et al., 2017; Cai et al., 2019; Zhou et al., 2021), which makes it suitable for the liquor brewing environment. *Monascus* excretes a variety of hydrolytic enzymes (saccharification enzymes, proteases, esterase, etc.) to improve the brewing quality of *Baijiu* (Chen et al., 2015), but also produces abundant beneficial metabolites, such as *Monascus* pigments (red, yellow, and orange pigments) (Feng et al., 2012; Chen et al., 2015), gamma-aminobutyric acid (GABA) (Kusdiyantini et al., 2021; Song et al., 2021) and Monacolin K (MK) (Zhang et al., 2020), which have antimicrobial functions, lowering blood pressure, and lowering blood lipids properties, respectively. *Monascus* pigments may be the main reason for the phenomenon of “red heart” in the TQ. Unfortunately, some *Monascus* can also produce citrinin (CIT) with nephrotoxicity (Gu et al., 2021; Liu et al., 2021), and its safety in the brewing process also needs to be assessed, but its related reports in the wine-making industry are relatively rare (Srianta et al., 2014; Huang et al., 2019). Normally, *Monascus* is often traditionally used in the brewing of yellow rice wine (Park et al., 2016; Huang et al., 2018). Nonetheless, it has also recently attracted attention from *Baijiu* researchers. For instance, Xu et al. (2020) isolated a good performance *Monascus* with a high ability to synthesize ethyl ester from the fermentation starter of *Baijiu*; Zhang et al. (2017) found that *Monascus* was positively correlated with glucose concentration, and played an important role in the starch saccharification in Chinese strong-aroma-type *Baijiu*. Based on the abovementioned literature reports, it is speculated that the cause of the red heart in TQ is the result of *Monascus* metabolism.

The annual production of LATB brewed with TQ is more than 70,000 tons. The plethora of microorganisms in TQ plays a crucial role in the process and determine the production of esters (ethyl acetate) (Fan et al., 2019), alcohols (ethanol, n-propanol, isobutanol, isoamyl alcohol, etc.) (Gerong et al., 2019; Hu et al., 2021b), and acids (acetic acid) (Liu and Sun, 2018; Xu et al., 2021). Additionally, compared with sensory evaluation standards, the assessment of microorganisms is more scientific and operable to reflect the quality of TQ. Currently, there are still many research gaps about TQ microorganisms. For example, the structure and function of microbiota in TQ and their effects on the flavor components of the original liquor are still poorly understood. Thus, addressing these issues should aid the improvement of quality for LATB.

In this study, two types of TQ [”green-covering” *Tuqu* with a non-red heart (NRH) and “green-covering” *Tuqu* with red heart (RH)] were selected to investigate the diversity of the microbial community through high-throughput sequencing (HTS). At the industrial scale, the effects of NRH and RH on the brewing quality of LATB were analyzed, revealing that the quality of the original liquor brewed by RH was better than that of NRH. Encouraged by this finding, and under the guidance of HTS data, two *Monascus* strains were isolated and identified from TQ samples, and we elucidated the characteristics of their metabolites. Interestingly, the results showed that citrinin was not detected in any samples, indicating that *Monascus* isolated from TQ was no safety risk, and the contents of GABA in the fermented grains of RH were higher than that of NRH during the fermentation. Based on this result, a stable RH production process was obtained by reinoculating different proportions of autochthonous *Monascus* bran seeds into the TQ-making process. Finally, combined with HTS and gas chromatography-flame ionization detection (GC-FID), the effects of the fortified strain *Monascus* on the fungal microbial composition in TQ and flavor substances in *Baijiu* were explored, yielding LATB with diverse flavor profiles and thereby enriching the potential aroma components of LATB. To our knowledge, this is the first comprehensive study to report the metabolic profiles of *Monascus* and its effects on the production of TQ and LATB. Together, our results provide additional guidance and excellent strain resources for establishing a more scientific evaluation standard for the quality of TQ and improving the quality of TQ.

## Materials and methods

### Sample collection

”Green-covering” *Tuqu* samples with “red heart” and “green-covering” *Tuqu* samples with non-red heart were collected in biological duplicates from the same *Tuqu*-making workshop in Daye, Hubei province, China. And the abovementioned samples were labeled as RH and NRH, respectively (Figure 1). These samples were stored at room temperature for brewing experiment and separation experiment, and some samples were stored at −20°C for DNA extraction.

### DNA extraction, amplification, and sequencing

Total genomic DNA of *Jiuqu* samples was extracted by using the cetyltrimethyl ammonium bromide (CTAB) or sodium dodecyl sulfate (SDS) method. After concentration and purification, extracted deoxyribonucleic acid (DNA) was diluted to 1 ng/μL using sterile water. Polymerase chain reaction (PCR) amplifications were carried out using ITS5-1737F (5′-GGAAGTAAAA GTCGTAACAAGG-3′) and ITS2-2043R (5′-GCTGCGTTCTT CATCGATGC-3′) (Schmidt et al., 2013), attaching a barcode to each sequence. All PCR reactions were conducted in 30 μL volumes, containing 15 μL of phusion high-fidelity PCR master mix (New England Biolabs, Beijing, China), 0.2 mM of forward and reverse primers, and ∼10 ng template DNA. PCR reactions were performed in triplicate, with the following cycling program: 98°C for 1 min, followed by 30 cycles of 98°C for 10 s, 50°C for 30 s, 72°C for 30 s, and 5 min at 72°C. PCR products with a bright band ranging from 200 to 400 bp for fungi were then mixed at equidensity ratios and purified with a GeneJET Gel Extraction Kit (Thermo Scientific, Waltham, MA, United States). High-throughput sequencing was performed on the Illumina HiSeq platform at Novogene Bioinformatics Technology Co., Ltd. (Beijing, China). All samples were performed in biological duplicates.

### Bioinformatics analysis

Paired-end reads from the original DNA fragments were merged using FLASH (version 1.2.7) (Magoč and Salzberg, 2011) and then assigned to samples based on the barcode. Sequences were analyzed with quantitative insights into microbial ecology (QIIME) (Caporaso et al., 2010), and inhouse Perl scripts were employed to determine alpha- and beta-diversity. First, reads were filtered by QIIME quality filters. Then we used pick_de_novo_otus.py to pick operational taxonomic units (OTUs) by making OTU table. Sequences with ≥97% similarity were assigned to the same OTUs using uparse software (UPARSE version 7.0.1001) (Edgar, 2013). Representative sequences for each OTU and used the RDP classifier (Wang et al., 2007) to annotate taxonomic information for each representative sequence. For the sequence data, they were deposited in the national center of biotechnology information (NCBI) database with accession number: PRJNA846056.^1^

### The production process of *Tuqu*

The *Guanyin* clay [73.8%, weight (w)], rice bran (24.6%, w), and ZQ (1.6%, w) were mixed, and the water content was kept at 32%. Next, the mixture was manually kneaded into a spherical billet with a diameter of approximately 8 cm. These spherical billets were placed in the box bed for the preculture at 27°C for 23 h. Then, the obtained billets were transferred to the steel shelf in another room for the second incubation, and the temperature of the culture room was controlled at 30°C. After cultivation for 7 days, the culture room was maintained at 35°C to reduce the water content of TQ to less than 10%. The natural production of TQ was performed in an open environment, and the traditional manual production process not only causes differences in the size and tightness of the TQ, but also leads to the differences in the organoleptic properties of TQ. Supplementary Figures 1A,B showed the fermentation temperature and moisture curves of the TQ’s production process. In addition, the production process of fortified TQ was as follows: the *Monascus* bran seeds were blended with TQ according to different weight proportions (0.3, 1.2, 2.4, 4.8, 7.2, and 9.6‰). The other steps were the same as those in the TQ production process.

### Industrial scale fermentation experiment

The grains were incubated in the water at 70–80°C for 20 h, after that the soaking water was filtered and steamed for 35 min under the pressure above 0.1 MPa. Next, the grains were covered with water and incubated for 5–20 min. After filtering them, the grains were steamed again for 30 min. Once the grains had cooled down, the experimental TQ was blended with grains at a weight ratio of 1:99 for saccharification overnight at 23°C. The sterilized previous industrial distilled grains were cooled and mixed with the saccharified grains at a volume ratio of 1:4. Fermentation was carried out in the temperature-controlled room (around 22°C) for 14 days. Supplementary Figures 1C,D showed the saccharification and fermentation temperature curves of the TQ’s brewing process. After that, the fermented grains were distilled and evaluated by GC-FID.

### Volatile components analysis

The volatile components in original liquor were determined by GC-FID on the Agilent 7890B GC system. The original liquor sample (1 mL) was transferred to a sterile 2 mL glass vial containing 10 μL of the internal standard mix (2-ethyl hexanol 163.34 mg/L, 2-methyl-2-butanol 160.10 mg/L, *n*-amyl acetate 176.21 mg/L). GC was conducted with the following setting. The injector temperature was set at 250°C, and the split ratio was 30:1. The oven temperature was held at 35°C for 1 min, then raised to 70°C at a rate of 3°C/min, finally raised to 190°C at a rate of 3.5°C/min, and maintained to 190°C for 25 min. Nitrogen (purity ≥ 99.999%) as the column carrier gas was at a constant flow rate of 1 mL/min. Volatile compounds were identified by matching with the retention time of standard materials. The quantitative analysis of volatile compounds was performed by comparison with the area of the internal standard. All the analytical sections have method validation followed by quantification. Ethanol content represents its volume in original liquor sample, which is detected *via* the portable density meter (DMA-35, 182 Anton Paar, Austria).

### Directional screening of *Monascus via* culture-dependent method

The 1.0 g sample was evenly spread on the potato dextrose agar (PDA) medium, and 1 mL of sterilized lactic acid solution (50%) was added to cover the sample. And then they were incubated at 30°C. Next, the white and red colonies were continuously selected and transferred to the new PDA medium by drawing lines method (Zhou et al., 2021).

### Preparation of *Monascus* bran seeds

The *Monascus* strain was activated on PDA plates and incubated for 7 days at 30°C under aerobic conditions, and spores were washed off with sterile water to prepare the spore suspension, the concentration of which was adjusted to 1.0 × 10^6^CFU/mL with a haemocytometer. Then, the abovementioned fungal spore suspensions were inoculated at 6% (v/v) inoculum size into 3 L flasks containing 300 g of bran medium [bran, containing 70% (w/w) water] for incubation at 30°C for 8 days. Then, the cultures were dried in an oven at 40°C with moisture controlled at 4%, and crushed. Finally, *Monascus* bran seeds were stored at 4°C for further use.

### Determination of metabolites

The solid samples of CIT, GABA and MK, were ultrasonically extracted in 0.1 M hydrochloric acid solution, 70% methanol solvent, and 75% ethanol solvent at room temperature for 30 min, respectively. Then, they were centrifuged at 12,000 rpm at 4°C for 40 min. After filtering them, the supernatants were frozen until the chemical analyses were performed *via* reversed phase high performance liquid chromatography (RP HPLC). The liquor samples were used directly for HPLC detection. The detection method for GABA was that 10 μL of purification aliquots were tested by RP HPLC analysis (Agilent Zorbax SB-Aq column; 5 μm; 4.6 mm × 250 mm; evaporative light scattering detector; 10% CH_3_CN in H_2_O for 45 min; 0.8 mL/min, 30°C). For the quantification of GABA, a standard curve was made as shown below. The standard solutions of GABA with different concentrations (32.34, 40.42, 64.68, 80.85, 129.36, 161.70, and 323.40 mg/L) were made and took 10 μL standard series of solutions for HPLC analysis to construct a standard curve. The determination coefficient of the linear regression calibration curve was higher than 0.99. The MK was detected by RP HPLC [Agilent Zorbax SB-C18; 5 μm; 4.6 mm × 250 mm; 77% MeOH in H_2_O (0.03% phosphoric acid) for 40 min; 1.0 mL/min, 25°C] (Light industry standard of China QB/T 2847-2007). The CIT was detected by the same HPLC column [35% CH_3_CN in H_2_O (0.1% phosphoric acid) for 40 min; 1 mL/min, 30°C] (China national standard GB5009.222-2016).

### Physicochemical analysis

The 1.0 g *Jiuqu* sample was inoculated into the steamed rice, mixed well, covered by the bowl, and incubated at 30°C for 24 h. A blank control was set without inoculation. 10.0 g of saccharified mash were added into 100 mL purified water, shaking at 20°C at 140 rpm. After soaked for 30 min, the supernatant was taken for the measurement of reducing sugar by Fehling’s solution method. The saccharification rate represents the number of grams of glucose produced by saccharifying 100 g of rice per gram of *Jiuqu* for 24 h. The aforementioned saccharified mash was mixed with 300 mL of water, shaking well, and incubated for 72 h at 30°C. The fermented mash was distilled and then took 100 mL distillate for the detection of alcohol. According to the measured temperature and the value of the alcohol meter, these were converted to the alcohol content at 20°C. The alcohol content represents the fermentation rate.

### Laboratory-scale fermentation experiment

The glutinous sorghum was washed three times with sterile water and added to the warm water for incubation at 60°C overnight. After that, the soaked grains were filtered and put into a sterilizing steamer for initial steaming at 115°C for 10 min. Then, the grains were covered with water and incubated for at 80°C 5 min. After filtering them, the soaked grains were sterilized again at 111°C for 10 min. Once the re-steamed grains were cooled to 30°C, 1,000 g of grains were transferred to a sterile ziplock bag. Next, 10 g of the experimental TQ were added to each bag. After thorough mixing, the bags were covered with a sterile wet towel filter and put into the incubator for saccharification at 30°C for about 24 h. Next day, the previous distilled grains were weighed and sterilized at 121°C for 30 min. Meanwhile, the content of each bag after saccharification was thoroughly mixed and half (about 500 g) was removed from the bag. Once the grains from the previous distillations were cooled to room temperature, the same weight (about 500 g) of these grains was added to each sterile gas sampling bag. The oxygen of all bags was removed by a water pump before closing the exhaust valve with a water lock. All bags were incubated at 30°C for 7 days. After the fermentation, the fermented grains were put into a special conical flask for distillation, and the first 100 mL of the distilled liquor was taken for the chemical analysis. Three biological replicates per TQ were performed.

### Statistical analysis

Origin Pro 2018 was used to draw the histogram of microbial abundance. Spearman’s correlation analysis was performed to explore the correlations between microorganisms and microorganisms or aroma components using the “dplyr”, “picante”, “reshape2” packages of R (version 4.0.3), and the correlation network was visualized with | ρ| > 0.8 and *p* < 0.05 in Cytoscape (version 3.7.1). The principal component analysis (PCA) of the built dataset was performed by SIMCA software (version 13.0). Significant differences were tested using one-way analysis of variance (ANOVA) in SPSS.

## Results

### The microbial diversity in non-red heart *Tuqu* and red heart *Tuqu*

According to the HTS data, the total effective sequence of the ITS1 rDNA gene was 310729. All the effective sequences were assigned to a total of 201 fungal operational taxonomic units (OTUs) at 97% sequence similarity. The alpha diversity indexes among two different *Jiuqu* samples (NRH and RH) were analyzed to assess the fugal community richness and diversity (Supplementary Table 1). The larger were the values of Chao1 or ACE indexes the higher was the community richness of the sample. A higher Shannon or Simpson index value indicated a higher community diversity of the sample. The Chao 1 indexes of NRH and RH were 176.97 and 325.11, respectively, and the Shannon indexes were 2.71 and 1.66, respectively. The Shannon index of RH was relatively smaller, which means that the fungal diversity was lower.

**FIGURE 2:**
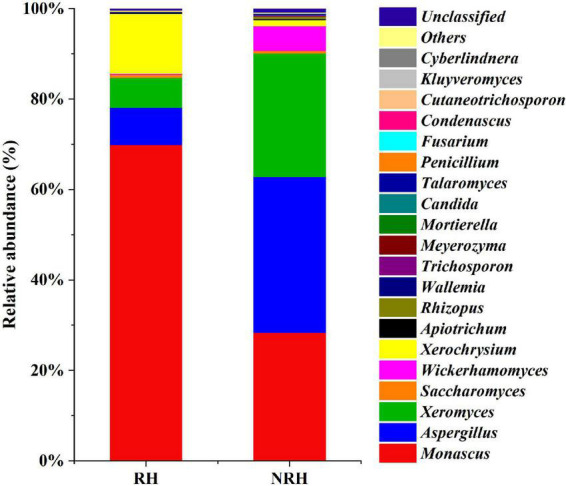
Fungal community composition in two *Jiuqu* samples. At the genera level, among the top 20 genera with relative abundance were greater than 0.1%.

In addition to the differences in alpha diversity among the samples, the relative abundances of fungal species detected in the two *Jiuqu* samples were also different (Figure 2). In all samples, the compositions of dominant genera were roughly the same, but the relative abundance of genera was different. The relative abundance of *Monascus* and *Aspergillus* was higher among the two types of *Jiuqu*. In RH, the main genera were *Monascus* (69.94%), *Xerochrysium* (13.23%), *Aspergillus* (8.22%), and *Xeromyces* (6.66%), while *Aspergillus* (34.44%), *Monascus* (28.41%), *Xeromyces* (27.31%) and *Wickerhamomyces* (5.50%) were identified as dominant genera in NRH. Interestingly, as an ester-producing yeast, *Wickerhamomyces* had a higher relative abundance in NRH than in RH, which indicated that the presence of *Monascus* may have a certain inhibitory effect on the growth of *Wickerhamomyces.*

### Evaluation of red heart *Tuqu* and non-red heart *Tuqu* at industrial scale light-aroma-type *Baijiu*

To further assess the fermentation performance of TQ with different sensory characteristics, RH and NRH were tested in septuplicate in an industrial scale sorghum-based fermentation. Interestingly, RH had a significant effect to improve the content of esters and reduce higher alcohols compared to NRH (Table 1), which were beneficial for LATB production. The content of ethyl acetate increased by 11.99% (*p* < 0.05) and higher alcohols decreased by 7.89% (*p* < 0.05). Additionally, there were no significant differences in the production of acetaldehyde and methanol. Furthermore, the average alcohol yield of the RH group decreased slightly compared with NRH by 1.35% (*p* < 0.05), indicating that RH affected the alcohol yield to a small extent.

**TABLE 1:** Characteristics of *Tuqu* with red heart (RH) and *Tuqu* with non-red heart (NRH) as measured in the industrial scale fermentations of light-aroma-type *Baijiu.*

Inoculum	Ethanol yield (%)*^a^	Acetaldehyde (mg/L)	Methanol (mg/L)	Ethyl acetate (mg/L)*	Higher alcohols (mg/L)*
RH	59.58 ± 0.57	314.84 ± 22.08	105.44 ± 10.78	1,153.47 ± 119.97	2,076.32 ± 117.18
NRH	60.39 ± 0.72	315.71 ± 22.99	107.14 ± 13.80	1,030.00 ± 61.37	2,254.28 ± 157.14

*p < 0.05. ^*a*^Ethanol yield represents the weight of 60% volume fraction of ethanol divided by the weight of grain.

### Isolation of microorganisms and analysis of microbial metabolites

Because the HTS data of RH revealed that the abundance of *Monascus* was the highest (69.94%), and that of the NRH was 28.41% (Figure 2), encouraged by this finding, we selected the sample of RH and NRH for the isolation of *Monascus.* Two types of *Monascus* strains, M1 (*Monascus purpureus*) and M2 (*Monascus ruber*), were isolated and identified by culturomics and microbiomics methods. According to previous reports, *Monascus* can produce some pigments (red, yellow, and orange pigments) (Chen et al., 2015), which may be the main reason for the phenomenon of “red heart” in the TQ. In addition, *Monascus* can produce various metabolites, such as GABA and MK, which can lower the blood pressure and lipid levels, respectively, but CIT can cause teratogenicity and mutagenicity. Therefore, to determine the characteristics of the metabolites in isolated *Monascus*, the fungus was fermented in a rice solid fermentation culture to prepare *Monascus-*fermented rice. Meanwhile, RH, NRH and their fermented grains and original liquor were also detected (Table 2). The obtained results showed that the two strains could produce GABA, and the production of M1 was significantly higher than that of M2. Table 2 shows that the content of GABA gradually increased during the fermentation process, and the content reached the maximum value at the end of fermentation. The cumulative content of GABA in the RH was slightly higher than that in NRH during the fermentation. In addition, no MK or CIT were detected in any of the samples, indicating that there was no safety risk of CIT in the whole fermentation process of LATB. Therefore, to investigate the effect of *Monascus* on the production of TQ, the autochthonous *Monascus* M1 was selected for fortified fermentation due to its high production of GABA and high enzyme activity (protease activity was 78.89 U/g, saccharification enzyme activity was 9,533.46 U/g⋅h, and esterase activity was 5.18 mg/g⋅100 h) (Supplementary Table 2). Furthermore, *Monascus* M1 can grow in a solid medium with a concentration of 23% ethanol, withstand a high temperature of 45°C, and endure relatively low pH (Supplementary Table 3). Therefore, *Monascus* M1 meets the requirements of the LATB brewing environment, and has the potential to improve the quality of liquor.

**TABLE 2:** Contents of gamma-aminobutyric acid (GABA) of *Monascus-*fermented rice, the fermented grains, and original *Baijiu.*

Sample	Time		GABA (mg/kg)
*Monascus-*fermented rice	M1		92.52 ± 9.81
	M2		10.47 ± 2.66
Fermented grains	NRH	0 day	5.62 ± 0.18
		4 days*	58.18 ± 0.67
		7 days *	88.23 ± 0.55
	RH	0 day	5.37 ± 0.32
		4 days *	71.63 ± 0.70
		7 days *	96.00 ± 0.90
Original liquor	NRH		–
	RH		–

”–” means not detected. *p < 0.05.

### Effects of fortified *Monascus* strain on volatile compounds of light-aroma-type *Baijiu*

To evaluate the effect of *Monascus* on TQ-making and flavor components of LATB, the selected strain M1 was inoculated for fortified TQ-making. First, M1 was prepared into bran seeds, and added to the production of TQ according to different weight proportion (0.3, 1.2, 2.4, 4.8, 7.2, and 9.6‰). The produced TQ with different levels of red heart phenomenon were named as experimental “red heart” TQ (ERH) and labeled as ERH1-6, respectively. TQ without fortified M1 strain with non-red heart, named as NRH1, was served as the control group (CK) (Supplementary Figure 2).

The saccharification rate and fermentation rate of TQ are important indicators to judge the quality of *Jiuqu*. As shown in Table 3, when the addition amount of *Monascus* bran seeds was large (ERH5 and ERH6), it brought negative disturbance of saccharifying and fermenting ability, the saccharification rate was low while the fermentation rate was too low to be detected. That meant adding more *Monascus* bran seeds in the TQ-making process could seriously affect the saccharification and fermentation rates of the TQ. However, the adding amount was 1.2‰ (ERH2), the rates of saccharification and fermentation reached maximum. Therefore, based on the control standard of quality, four groups (ERH1, ERH2, ERH4, and NRH1) were selected for brewing to explore the effect of ERH on the flavor compounds of LATB.

**TABLE 3:** Saccharification and fermentation rates of *Tuqu* without fortified M1 strain with non-red heart (NRH1) and experimental “red heart” *Tuqu* (ERH).

Samples	Saccharification rate (g/100 g)	Fermentation rate (%)
NRH1	26.96 ± 0.40	32.33 ± 0.40
ERH1	19.40 ± 0.62	34.26 ± 0.50
ERH2	20.13 ± 0.32	35.26 ± 0.41
ERH3	3.80 ± 0.26	29.8 ± 0.30
ERH4	15.96 ± 0.20	35.26 ± 0.30
ERH5	1.60 ± 0.40	–
ERH6	1.80 ± 0.20	–

”–” means too low to be detected. The low saccharification rate in sample ERH3 was due to technical problems in the TQ production process.

**FIGURE 3:**
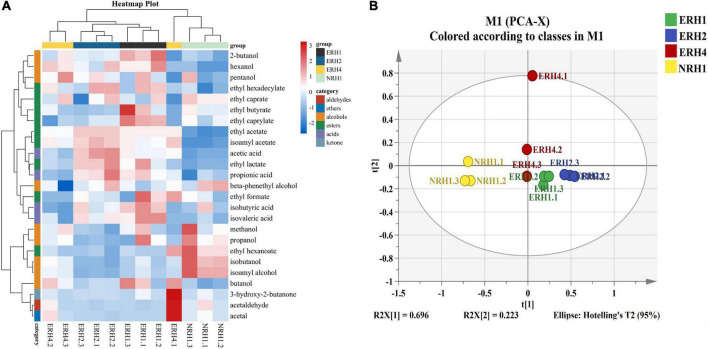
**(A)** Heatmap of flavor metabolites and hierarchical clustering. Flavor compounds were transformed by z-score. Clustering analysis was performed using the Pearson correlation coefficient and euclidean distance based on the flavor contents in the *Jiuqu* sample. **(B)** Principal component analysis (PCA) analysis of flavor compounds in original liquor fermented by 4 *Tuqu.*

The main volatile compounds of LATB included five esters, eight alcohols, four acids, and one aldehyde were detected by GC-FID (Figure 3A). PCA showed the metabolic profiles in different TQ (Figure 3B), the cumulative variance contribution of principal components R2X(1) (0.696) and R2X(2) (0.223) was 0.919 (>0.8), which could reflect the main characteristics of TQ samples. The ERH group was clustered together, far away from the NRH1 group, indicating that the ERH had a significant effect on the flavor profiles of original liquor. In addition, ERH1, ERH2, and ERH4 groups were situated in different places, showing that the effect of fortified TQ with different proportion of *Monascus* strain on flavor components was remarkably different. Hence, both heat-map and PCA showed that it’s a potential and effective method to improve the quality of fermentative starter by fortifying the appropriate proportion of selected strains during TQ preparation.

The volatile components contents brewed by different *Jiuqu* were showed in Supplementary Table 4. The esters in the original liquors of ERH were mainly ethyl acetate and ethyl lactate, the total esters (2,861.10–3,439.66 mg/L) were about two times more than that of NRH1 (1,535.15 mg/L). Furthermore, ethyl acetate is the key flavor substance in LABT (Fan et al., 2019), the content of ethyl acetate in ERH increased by 96.11–123.93% compared with NRH1. ERH2 group had the highest contents of ethyl acetate (2,901.29 mg/L) and ethyl lactate (442.74 mg/L). The higher alcohols can make the liquor taste harmonious, give people a mellow feeling at appropriate concentrations, and it will produce unpleasant bitter taste, headache after drinking at high concentrations (Gerong et al., 2019; Hu et al., 2021b). The higher alcohols in ERH groups mainly were n-propanol, isobutanol, and isoamyl alcohol. Compared with NRH1 group, the content of higher alcohols in the ERH group was reduced by 15.39–27.64%. Among these, the content of higher alcohols in the ERH2 group was the lowest (1,276.48 mg/L). There were significant differences in the content of *n*-propanol (decreased by 0.98–14.03%), isobutanol (decreased by 21.66–36.57%), and isoamyl alcohol (decreased by 13.73–27.23%) in the ERH group compared with NRH1 group. Acetaldehyde is also a vital compound which contributes to the aroma of LATB at low concentrations, and it can negatively influence the overall flavor balance of LATB at high concentrations. Therefore, lower acetaldehyde is considered a positive role for LATB production (Chen et al., 2022). The results showed that the ERH with relative low addition of *Monascus* (ERH1 and ERH2) had a better reduction effect on acetaldehyde (decreased about 78%). However, when the addition of *Monascus* was greater (ERH4), the contents of acetaldehyde increased. In addition, a significant increase in the content of acetic acid was observed in all the fortified TQ fermentation samples, especially for the sample ERH2. The content of acetic acid in NRH1 was 262.93 mg/L, whereas, the acetic acid in ERH2 reached 641.96 mg/L, which was an increase of nearly 144.15%. As for the alcohol yield, the ERH group decreased by 1.61–6.20% compared with the NRH1 group, indicating that TQ inoculated with the *Monascus* strain could have a slight impact on the formation of alcohol.

### Effects of fortified *Monascus* strain on the microorganisms of *Jiuqu*

**FIGURE 4:**
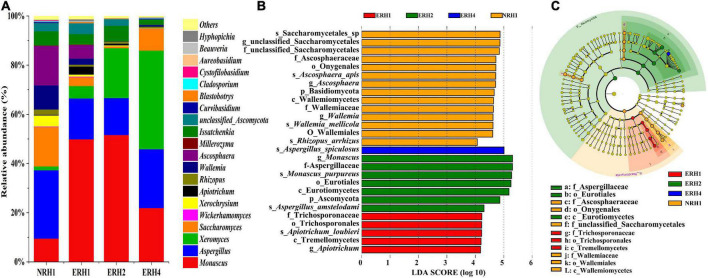
**(A)** Fungal community compositions in *Tuqu* without fortified M1 strain with non-red heart (NRH1) and experimental “red heart” *Tuqu* (ERH). At the genera level, among the top 20 genera with relative abundance were greater than 0.1%. Linear discriminant analysis (LDA) effect size (LEfSe). **(B)** Fungal taxa that showed significantly different abundances for samples. **(C)** Taxonomic cladogram of the discriminant analyzed by LEfSe. Rings from the inner to the outer portion of the graph represent taxonomic ranks from phylum to genus. The nodes in each taxonomic rank were defined at the same level of classification and the sizes of the nodes were proportional to their relative abundances. Nodes with a lime color represented no significant variation in the abundances of the taxa. Areas highlighted with the additional different colors represented different groups, and the nodes in the additional different colors distinguished the different samples. The same color nodes in the branches were defined as significant different taxon biomarkers representing the distinguishing taxa in the same color group.

The relative abundance of different fungal microorganisms at the genus level of ERH and NRH1 samples is shown in Figures 4A. The fungal microorganisms were similar, and the dominant fungi were *Monascus*, *Xeromyces*, *Saccharomyces*, *Aspergillus*, *Ascosphaera*, *Issatchenkia*, and *Wallemia* in all samples. Interestingly, when the added ratio of *Monascus* bran seeds increased, the abundance of *Monascus* in ERH did not increase. *Monascus* was the most abundant genus in ERH2, and the least abundant in NRH1, followed by ERH4. The abundance of *Xeromyces* increased, and the richness of *Ascosphaera* and *Wallemia* decreased with the increase in *Monascus* bran seeds addition. *Aspergillus* and *Saccharomyces* were relatively more abundant in NRH1, followed by ERH4, and less abundant in ERH1 and ERH2. The abundances of the detected fungal taxa in the samples were also analyzed using the linear discriminant analysis (LDA) effect size (LEfSe) method. LDA highlighted that a total of 27 fungal taxa were different in all samples by the statistically significant LDA threshold of >4 (Figure 4B). These differentiating taxa were shown on the phylogenetic tree of the total fungal community from the phylum to the species level using LEfSe (Figure 4C). The four groups displayed differentially abundant fungi including two phyla, three classes, four orders, five families, five genera, and eight species. Notably, the abundance of *Aspergillus* was statistically different and distinctive in ERH4. NRH1 had the largest number of distinguishing taxa, including *Ascosphaera*, *Wallemia*, and unclassified *saccharomyces*. The NRH1 variety was also the most rich in low-abundance fungal communities, such as the genera of *Ascosphaera* and *Wallemia.* The abundance of *Monascus* was significant in ERH2 compared with that in other samples.

### Correlation analysis between microorganisms and flavor compounds

**FIGURE 5:**
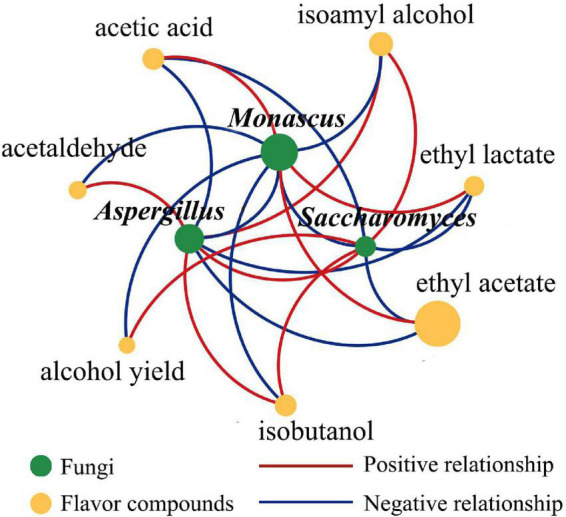
Correlation network diagram among dominant fungal genera and flavor compounds. Microorganisms and flavor compounds were represented by green circle and yellow circle modules, respectively, the size of the circle represented the relative amount of microorganisms and flavor compounds; positive and negative relationships were represented by red solid and blue solid, respectively.

The correlations between the microorganisms in *Jiuqu* and the volatile compounds of the original liquor were analyzed *via* Cytoscape, with | ρ| > 0.8 and *p* < 0.05 to further explore the effect of fortified TQ on fermentation quality, the main correlations were shown in Figure 5. *Monascus* had a positive effect on the production of ethyl acetate, ethyl lactate, and acetic acid and had a negative effect on the production of isobutanol, isoamyl alcohol, acetaldehyde, and alcohol. These results were analogous to those reported in the literature (Fang, 2013). *Saccharomyces* had a positive effect on isobutanol, isoamyl alcohol, and alcohol, which was consistent with the study that showed that higher alcohols were mainly synthesized by yeast. *Aspergillus* could promote the production of isobutanol, isoamyl alcohol, and acetaldehyde. Additionally, *Monascus* could inhibit the growth of *Saccharomyces* and *Aspergillus*, while *Aspergillus* and *Saccharomyces* could promote the growth of each other. Interestingly, the relative abundance of *Xeromyces* was higher (1.55–40.09%), but it had no correlation with most microorganisms and no significant promotion or inhibition with flavor substances.

## Discussion

With the improvement of people’s living standards, people’s requirements for product quality are increasing, and traditional *Baijiu* should have better taste and health implications. TQ, as a complex microbial cocktail, can provide rich microbial resources and flavor prerequisite substances for LATB, which determines the quality of *Baijiu*. In recent years, the study of TQ has attracted considerable attention. Previous research often focused on yeasts and *Rhizopus* (Wang et al., 2020; Chen et al., 2022). Little attention was given to the influence of *Monascus* on *Jiuqu*. *Monascus* can produce a variety of hydrolytic enzymes, such as saccharification enzymes, proteases, and esterases, and has higher acid and alcohol tolerances, which meets the requirements of the LATB brewing environment. Moreover, *Monascus* can produce a large number of beneficial secondary metabolites, such as GABA, MK, and ergosterol, etc., which boost the healthy factors of *Baijiu*. *Monascus* has been encountered in some distilleries, and it has been found to improve the contents of ethyl hexanoate and ethyl acetate during the *Baijiu* production (Feng et al., 2010; Liu et al., 2012). Surprisingly, there is no relevant report on how *Monascus* affects the quality of TQ and flavor compounds of LATB.

To our knowledge, in this project, systematic and intensive research on the application of *Monascus* in LATB was conducted for the first time, analysing the metabolism of *Monascus*, and clarifying the effect of RH on the quality of LATB. The correlation between the microorganisms in *Jiuqu* and the volatile compounds of the original liquor was analyzed. The microbial differences in samples were studied *via* HTS and culture-dependent methods. HTS data showed that *Monascus* was abundant in RH and NRH (Figure 2). HTS-directed separation of cultivable microorganisms from TQ samples resulted in the isolation and identification of two species of *Monascus.* Because TQ was produced manually, there were differences in size and tightness that could lead to internal differences in TQ. Chen et al. (2020) reported that the acidity and moisture content in the heart of *Jiuqu* were higher than those on the surface. Meanwhile, *Monascus* prefers acidity and sufficient water (Said et al., 2010) and can resist low oxygen (Cai et al., 2019), thus, *Monascus* can grow and multiply inside the TQ, and *Monascus* secretes large amounts of pigments, forming the phenomenon of a “red heart”, which was verified by fortified TQ-making experiments. Then, the “red heart” phenomenon of TQ is more obvious and more stable. Therefore, *Monascus* causes the “red heart” of TQ.

The results of the brewing experiment using ERH demonstrated that *Monascus* significantly increased the production of ethyl acetate and acetic acid, and decreased the production of higher alcohols and acetaldehyde, which were beneficial to the quality of LATB. According to the sensory evaluation results, the *Baijiu* produced by RH had a better taste than that produced by NRH. The fortified results confirmed the empirical knowledge that RH was a high-quality ***Jiuqu***. After a long period of selection and domestication, ***Jiuqu*** microorganisms have evolved to be compatible with each other, forming a stable ecosystem (Li et al., 2014). Therefore, it is important to avoid the side effects produced by fortified strains. In previous research, exotic strains were usually mixed with ***Jiuqu*** to make fortified fermentative starters, which may bring the risk of destroying the original ecosystem. For instance, Nie et al. (2015) found that the introduced non-***Jiuqu***-original yeast strains were easily inhibited or killed by the indigenous yeast strains and other autochthonous inhabitants in ***Jiuqu***, or even worse, the ***Jiuqu*** aboriginal yeasts may be killed by the introduced microorganisms. In our study, the ERH was produced by reinoculating ***Monascus*** isolated from TQ in the TQ-making process. Compared with previous studies, the developed strategy showed some unique features. First, the selective indigenous strains had no safety risk. Second, they could easily adapt to the local environment and did not produce undesired substances. Hence, an increase in desired compounds and a decrease in undesired compounds can be obtained by fortifying indigenous strains.

The next step was to explore the effect of the addition ratio of *Monascus* bran seeds on the brewing quality of LATB. It was found that when more *Monascus* bran seeds were added, a negative impact on the quality of TQ and original liquor occurred. In contrast, when an appropriate amount of *Monascus* bran seeds was added, TQ had a greater beneficial effect on *Baijiu* quality. This result was beneficial for the mass production of RH, which could reduce the amount of *Monascus* bran seeds and produce better LATB. Compared to that of CK, the optimized addition ratio was 1.2‰ by weight (ERH2). *Monascus* abundance was characteristic of sample ERH2 (Figure 4B), which increased the production of ethyl acetate and ethyl lactate by 123.93 and 162.37%, respectively. These were the important compounds that contributed to the overall flavor balance and were perceived as fragrant in LATB. Previous reports showed that esterases produced by different *Monascus* had high specificity and could catalyze the hexanoic acid and ethanol to generate ethyl hexanoate (Chen et al., 2011). For example, in the study of Xu et al. (2020), *Monascus* had a strong promoting effect on the esterification of ethyl hexanoate and a weak effect on the catalysis of ethyl lactate, ethyl acetate, and ethyl butyrate, indicating that the esterases of *Monascus* were the enzymes with strong specificity. However, in our project, there were no significant differences in the content of ethyl hexanoate of *Baijiu* fermented using ERH compared to CK; however, the content of ethyl acetate and ethyl lactate significantly increased. This may be due to the different high esterase activity produced by *Monascus* and microbial interactions, as well as the complex brewing process of the LATB that provided a large amount of acetic acid, lactic acid, and ethanol for *Monascus*, leading to the catalytic formation of ethyl acetate and ethyl lactate. Thus, it showed that the esterase characteristics of *Monascus* isolated from diverse environments were significantly different. Their growth under different fermentation conditions could stimulate the production of different biological esterases, resulting in the generation of different esters.

The obtained results of HPLC detection showed that no citrinin was detected in the fermented grains and *Baijiu* samples, demonstrating no safety risk concerns to workers and consumers. *Monascus* can secrete highly active glutamate decarboxylase to synthesize GABA (Gong et al., 2019; Ye et al., 2021), which is considered to be an active substance for lowering blood pressure. In this project, we found that the GABA content of RH was slightly higher than that of the NRH during the brewing process, verifying RH possessed higher health values. Guo et al. (2021) tested 20 Chinese *Baijiu* samples by experimental advanced method and found that the highest concentration of GABA in *Baijiu* samples was 0.867 mg/L, but GABA was not detected in our *Baijiu* samples, which may be related to our distillation technology, or the content was too low to be detected. In addition, MK produced by *Monascus* fermentation could effectively inhibit 3-hydroxy-3-methyl glutaryl coenzyme A (HMG-CoA, a key enzyme in cholesterol biosynthesis) reductase activity, which in turn regulated blood lipid levels (Zhang et al., 2020). In this work, MK was not detected in the fermented grains or *Monascus* productions, possibly due to its minute contents being below the detection limit of the method used.

When functional microorganisms were used to enhance *Baijiu* quality, in addition to analyzing individual functional strains, it was also necessary to explore their impact on the overall microbial community structure during the brewing process (Chen et al., 2020). The microbial interactions affected the entire microbial growth and fermentative quality (Wu et al., 2014). In our experiment, the microbial species and the main fermentation properties in TQ were maintained before and after *Monascus* inoculation. HTS data and correlation network analysis results showed that the dominant fungi in ERH and NRH1, including *Monascus*, *Saccharomyces*, and *Aspergillus*, had a certain balance relationship. Properly speaking, *Monascus* inhibited the growth of *Saccharomyces* and *Aspergillus* to a certain degree, while the growth of *Saccharomyces* and *Aspergillus* could promote each other. The possible mechanism behind this phenomenon was that *Monascus* can secrete a variety of secondary metabolites, which may inhibit the growth of *Saccharomyces*. Martínková et al. (1995) reported that orange pigment produced by *Monascus* could inhibit the growth of *Saccharomyces.* This was supposed to be one of the reasons for the lower higher alcohols production in the original liquor produced by ERH, because the higher alcohols in *Baijiu* were mainly produced by yeast through the Ehrlich and Harris pathways (Cheng et al., 2011; Wang et al., 2020). Yuan and Chen (2021) found that *Aspergillus niger* significantly inhibited the growth of *Monascus* when they were cocultured, and Wang et al. (2012) found that yellow pigment produced by *Monascus* strongly inhibited the spore formation of *A. niger*. Both *Monascus* and *Aspergillus* were dominant genera in TQ, and they competed with each other for nutrients and growth space, demonstrating that the balance between different microbial species was crucial for flavor substances in LATB. Therefore, it was an effective and systematic strategy to improve the quality of LATB, inoculating the autochthonous *Monascus* in TQ with proper proportion.

In contrast, other microorganisms were not flavor compound producers, but they showed activity when co-occurred with those flavor-producing microorganisms, which was also important to shape the community structure and function (Wang et al., 2019). For example, *Pichia membranifaciens* and *Bacillus amyloliquefaciens* were not efficient flavor producers, and their addition alleviated competition among *Saccharomyces cerevisiae*, *Issatchenkia orientalis*, and *Bacillus licheniformis*, simultaneously altering their growth rates and flavor production (Wu et al., 2014). In our experiment, *Xerormyces* had the ability to grow in a low water environment (Leong et al., 2011), and it was not an efficient flavor compound producer. The relative abundance of *Xeromyces* was higher in ERH (5.03–40.09%) than in NRH1, and the abundance of *Xeromyces* increased with the addition of *Monascus* bran seeds. However, *Xeromyces* had no correlation with most microorganisms and had no significant promoting or inhibiting effects on the production of flavor substances (Figure 5). This phenomenon occurs possibly because *Xeromyces* alleviated the competition among flavor compound producers (*Aspergillus*, *Monascus*, and *Saccharomyces*) and maintained the stability of microbiota structure and microbial dynamics during TQ-based spontaneous LATB fermentation.

## Conclusion

In conclusion, it is the metabolites of *Monascus* that cause the “red heart” of TQ, and there was no safety risk of CIT in LATB fermented with TQ. In addition, the cumulative content of GABA in the fermented grains of RH was slightly higher than that in NRH during the fermentation. Fundamentally, RH had a positive effect on *Baijiu* quality compared to NRH. After studying the microorganisms in TQ and the flavor substances of LATB by optimizing the inoculation ratio of *Monascus* bran seeds in TQ, it was found that the influence of inoculated *Monascus* on the quality of *Baijiu* was achieved by affecting the overall microbial community structure. Under the optimal fortified ratio, the contents of ethyl acetate and ethyl lactate were increased, and the productions of acetaldehyde and higher alcohols were decreased. The obtained results provided unique fungal resources for screening superior strains and improving the quality of TQ and the original liquor. In the future studies, the inoculation ratio of *Monascus*, production process parameters of TQ, and detection method of functional components will be continuously optimized, which will improve the intrinsic health value of *Baijiu*.

## Data availability statement

The datasets presented in this study can be found in online repositories. The names of the repository/repositories and accession number(s) can be found in the article/Supplementary material.

## Author contributions

LiZ, LC, LeZ, and GZ participated in experimental processes. LiZ and LL wrote the manuscript. LL, GZ, BL, and ZZ helped to modify the figures and tables. SC, QY, and JT provided the assistance and guidance throughout the research. SC assisted the manuscript checking. All authors contributed to the article and approved the submitted version.

## Footnotes

1
http://www.ncbi.nlm.nih.gov/

## References

[B1] Cai Q. Q. Zhou K. X. Liu Z. B. Zhang C. Zhang W. Lv X. (2019). Studies on growth inhibitory factors of *Monascus* in the brewing process of Hongqu rice wine. *J. Chin. Inst. Food Sci. Technol.* 10 141–149.

[B2] Caporaso J. G. Kuczynski J. Stombaugh J. Bittinger K. Bushman F. D. Costello E. K. (2010). QIIME allows analysis of high-throughput community sequencing data. *Nat. Methods* 7 335–336. 10.1038/nmeth.f.303 20383131 PMC3156573

[B3] Chen M. B. Liu H. Zhen D. Fang S. L. (2011). Research on the esterification property of esterase produced by *Monascus* sp. *Afr. J. Biotechnol.* 10 5166–5172. 10.1186/1471-2180-11-134 21672225 PMC3130646

[B4] Chen S. X. Perez S. G. Herrera M. B. Zhu L. P. Liu Y. C. Steensels J. (2022). Breeding of New Saccharomyces cerevisiae hybrids with reduced higher alcohol production for Light-aroma-type-Xiaoqu Baijiu production. *J. Am. Soc. Brew. Chem.* [Epub ahead of print]. 10.1080/03610470.2022.2033608

[B5] Chen W. P. He Y. Zhou Y. Shao Y. C. Chen F. S. (2015). Edible filamentous fungi from the species *Monascus*: early traditional fermentations, modern molecular biology, and future genomics. *Compr. Rev. Food Sci. Food Saf.* 14 555–567. 10.1111/1541-4337.12145

[B6] Chen Y. R. Li K. Liu T. Li R. Y. Fu G. Wan Y. (2020). Analysis of difference in microbial community and physicochemical indices between surface and central parts of Chinese Special-flavor Baijiu Daqu. *Front. Microbiol.* 11:592421. 10.3389/fmicb.2020.592421 33519730 PMC7840566

[B7] Cheng J. Qin W. Zhao X. (2011). Formation mechanism and regulation of higher alcohols in wine brewing. *Chinese Brew.* 12 9–11.

[B8] Edgar R. C. (2013). UPARSE: highly accurate OUT sequences from microbial amplicons reads. *Nat. Methods* 10 996–998. 10.1038/nmeth.2604 23955772

[B9] Fan W. Xu Y. Qian M. (2019). Current practice and future trends of aroma and flavor research in Chinese Baijiu. *Am. Chem. Soc.* 4 145–175. 10.1021/bk-2019-1321.ch012

[B10] Fang Y. J. (2013). The role of *Monascus* in Chinese liquor production. *Chinese Brew.* 4 133–135. 10.3969/j.issn.0254-5071.2013.04.030

[B11] Feng C. Wang G. Liang Y. Tian H. Wang L. (2004). Study on *Aspergillus clavulanum* in green-covered Guanyin Tuqu. *Liq. Making Sci. Technol.* 5 40–43. 10.3969/j.issn.1001-9286.2004.05.013

[B12] Feng X. S. Wang Y. Fu W. X. Meng Q. Y. (2010). Application of *Monascus* in the production of Xifeng wheat Daqu. *Liq. Making Sci. Technol.* 1 36–38. 10.3969/j.issn.1002-8110.2010.01.014

[B13] Feng Y. L. Shao Y. C. Chen F. S. (2012). *Monascus* pigments. *Appl. Microbiol. Biotechnol.* 96 1421–1440. 10.1007/s00253-012-4504-3 23104643

[B14] Gerong Z. Huang F. J. Han X. L. Jiang W. Wang D. L. Liu S. (2019). A method for determining associations between drinking discomforts and key higher alcohols in strong-aroma Baijiu. *Food Ferment. Ind.* 14 191–195. 10.13995/j.cnki.11-1802/ts.019118

[B15] Gong J. Y. Wang J. J. Jin Y. X. Xiao G. N. (2019). Effect of γ-aminobutyric acid supplementation on the composition of Chinese rice wine. *J. Inst. Brew.* 125 110–117. 10.1002/jib.539

[B16] Gu S. Chen Z. Wang F. Wang B. L. (2021). Characterization and inhibition of four fungi producing citrinin in various culture media. *Biotechnol. Lett.* 43 701–710. 10.1007/s10529-020-03061-2 33386497

[B17] Guo Y. Li J. H. Li X. X. Li Y. K. Wang G. M. (2021). Determination of γ-aminobutyric acid and nucleosides in Chinese Baijiu by HPLC. *Food Res. Dev.* 42 6–12. 10.12161/j.issn.1005-6521.2021.01.026

[B18] Hu Y. L. Wang L. Y. Zhang Z. J. Yang Q. Chen S. X. Zhang L. (2021a). Microbial community changes during the mechanized production of light aroma Xiaoqu Baijiu. *Biotechnol. Biotechnol. Equip.* 35 487–495. 10.1080/13102818.2021.1892525

[B19] Hu Y. L. Yang Q. Chen D. Fu B. Zhang Y. Zhang Y. (2021b). Study on microbial communities and higher alcohol formations in the fermentation of Chinese Xiaoqu Baijiu produced by traditional and new mechanical technologies. *Food Res. Int.* 140:109876. 10.1016/j.foodres.2020.109876 33648194

[B20] Huang Z. R. Guo W. L. Zhou W. B. Lu L. Xu J. X. Hong J. L. (2019). Microbial communities and volatile metabolites in different traditional fermentation starters used for HongQu glutinous rice wine. *Food Res. Int.* 121 593–603. 10.1016/j.foodres.2018.12.024 31108786

[B21] Huang Z. R. Hong J. L. Xu J. X. Lu L. Gou W. L. Pan Y. Y. (2018). Exploring core functional microbiota responsible for the production of volatile flavour during the traditional brewing of Wuyi Hong Qu glutinous rice wine. *Food Microbiol.* 76 487–496. 10.1016/j.fm.2018.07.014 30166178

[B22] Jin G. Y. Zhu Y. Xu Y. (2017). Mystery behind Chinese liquor fermentation. *Trends Food Sci. Technol.* 63 18–28. 10.1016/j.tifs.2017.02.016

[B23] Kusdiyantini E. Nurhayati Ferniah R. S. (2021). Production of γ-aminobutyric acid (GABA) by *Monascus purpureus* isolated from angkak, a mold isolated from angkak in semarang, indonesia. *J. Phys. Conf. Ser.* 1943:e012098. 10.1088/1742-6596/1943/1/012098

[B24] Leong S. L. Pettersson O. V. Rice T. Hocking A. D. Schnürera J. (2011). The extreme xerophilic mould *Xeromyces bisporus*-Growth and competition at various water activities. *Int. J. Food Microbiol.* 145 57–63. 10.1016/j.ijfoodmicro.2010.11.025 21145608

[B25] Li P. Li S. Cheng L. Luo L. (2014). Analyzing the relation between the microbial diversity of Daqu and the turbidity spoilage of traditional Chinese vinegar. *Appl. Microbiol. Biotechnol.* 98 6073–6084. 10.1007/s00253-014-5697-4 24691870

[B26] Li R. L. Fang S. L. Chen M. B. Liu H. (2010). Study on the activity of fungal glucoamylase in green-covered Guanyin Tuqu. *Liquor Making* 37 50–52. 10.3969/j.issn.1002-8110.2010.01.020

[B27] Liu C. L. Wei Y. C. Ren R. B. (2012). Three tips in the production of red core starter for Fenjiu. *Liq. Making Sci. Technol.* 6 73–75. 10.13746/j.njkj.2012.06.078

[B28] Liu H. L. Sun B. G. (2018). Effect of fermentation processing on the flavor of Baijiu. *J. Agric. Food Chem.* 66 5425–5432. 10.1021/acs.jafc.8b00692 29751730

[B29] Liu H. Q. Huang Z. F. Yang S. Z. Tian X. F. Wu Z. Q. (2021). Inducing red pigment and inhibiting citrinin production by adding lanthanum(III) ion in *Monascus purpureus* fermentation. *Appl. Microbiol. Biotechnol.* 105 1905–1912. 10.1007/s00253-021-11162-9 33576885

[B30] Magoč T. Salzberg S. L. (2011). FLASH: fast length adjustment of short reads to improve genome assemblies. *Bioinformatics* 27 2957–2963. 10.1093/bioinformatics/btr507 21903629 PMC3198573

[B31] Martínková L. Zlová P. J. Veselý D. (1995). Biological activity of polyketide pigments produced by the fungus *Monascus*. *J. Appl. Microbiol.* 79 609–616. 10.1111/j.1365-2672.1995.tb00944.x

[B32] Nie Z. Q. Zheng Y. Du H. F. Xie S. K. Wang M. (2015). Dynamics and diversity of microbial community succession in traditional fermentation of Shanxi aged vinegar. *Food Microbiol.* 47 62–68. 10.1016/j.fm.2014.11.006 25583338

[B33] Park K. H. Liu Z. B. Park C. S. Ni L. (2016). Microbiota associated with the starter cultures and brewing process of traditional Hong Qu glutinous rice wine. *Food Sci. Biotechnol.* 25 649–658. 10.1007/s10068-016-0115-6 30263319 PMC6049153

[B34] Said F. M. Chisti Y. Brooks J. D. (2010). The effects of forced aeration and initial moisturelevel on red pigment and biomass production by *Monascus ruber* in packed bed solid statefermentation. *Int. J. Environ. Sci. Dev.* 1 1–4. 10.7763/IJESD.2010.V1.1

[B35] Schmidt P. Bálint M. Greshake B. Bandow C. Römbke J. Schmitt I. (2013). Illumina metabarcoding of a soil fungal community. *Soil Biol. Biochem.* 65, 128–132. 10.1016/j.soilbio.2013.05.014

[B36] Song C. N. Zhu L. P. Shao Y. C. Chen F. S. (2021). Enhancement of GABA content in Hongqu wine by optimisation of fermentation conditions using response surface methodology. *Czech J. Food Sci.* 39 297–304.

[B37] Srianta I. Ristiarini S. Nugerahani I. Sen S. K. Zhang B. B. Xu G. R. (2014). Recent research and development of *Monascus* fermentation products. *Int. Food Res. J.* 21 1–12. 10.1186/1472-6963-13-16 23305316 PMC3549780

[B38] Tang J. Liu Y. C. Lin B. Zhu H. Jiang W. Yang Q. (2022). Effects of ultra-long fermentation time on the microbial community and flavor components of light-flavor Xiaoqu Baijiu based on fermentation tanks. *World J. Microb. Biot.* 38 1–14. 10.1007/s11274-021-03183-3 34817705 PMC8611178

[B39] Tian H. Feng C. (1999). Discussion on the optimization of the making of the green-covered Guanyin Tuqu. *Liq. Making Sci. Technol.* 1 24–25.

[B40] Wang Q. Garrity G. M. Tiedje J. M. Cole J. R. (2007). Naive Bayesian classifier for rapid assignment of rRNA sequences into the new bacterial taxonomy. *Appl. Environ. Microbiol.* 73 5261–5267. 10.1128/AEM.00062-07 17586664 PMC1950982

[B41] Wang S. L. Wu Q. Nie Y. Xu Y. (2019). Construction of synthetic microbiota for reproducible flavor metabolism in Chinese light aroma type liquor produced by solid-state fermentation. *Appl. Environ. Microbiol.* 85 1–18. 10.1128/AEM.03090-18 30850432 PMC6498162

[B42] Wang Y. W. Wu X. H. Wang H. (2012). Studies on the inhibitory effect and components of *Monascus*. *China Condiment.* 2 32–34. 10.3969/j.issn.1000-9973.2012.02.009

[B43] Wang Z. Su Z. X. Yang Q. Liu Y. C. Lin B. Chen S. (2020). Characterizing relationship of microbial community in Xiaoqu and volatiles of Light-aroma-type Xiaoqu Baijiu. *Food Sci. Technol. Res.* 26 749–758. 10.3136/fstr.26.749

[B44] Weusthuis R. A. Mars A. E. Springer J. Wolbert E. J. (2017). *Monascus ruber* as cell factory for lactic acid production at low pH. *Metab. Eng.* 42 66–73. 10.1016/j.ymben.2017.05.005 28583672

[B45] Wu Q. Ling J. Xu Y. (2014). Starter culture selection for making Chinese sesame-flavored liquor based on microbial metabolic activity in mixedculture fermentation. *Appl. Environ. Microbiol.* 80 4450–4459. 10.1128/AEM.00905-14 24814798 PMC4068675

[B46] Xu C. Sun B. Xu Y. Fan G. Li X. (2020). Identification of ethyl hexanoate synthesis esterifying enzymes production strain and its enzyme production conditions optimization. *J. Chin. Inst. Food Sci. Technol.* 5 138–147. 10.16429/j.1009-7848.2020.05.018

[B47] Xu Y. Q. Wang X. C. Liu X. Li X. T. Zhang C. N. Li W. (2021). Discovery and development of a novel short-chain fatty acid ester synthetic biocatalyst under aqueous phase from *Monascus purpureus* isolated from Baijiu. *Food Chem.* 338:128025. 10.1016/j.foodchem.2020.128025 32927200

[B48] Ye H. Wang J. Shi J. Du J. Y. Zhou Y. H. Huang M. (2021). Automatic and intelligent technologies of solid-state fermentation process of Baijiu production: applications, challenges, and prospects. *Foods* 3:680. 10.3390/foods10030680 PMC8004889 33806949

[B49] Yuan X. Chen F. S. (2021). Cocultivation study of *Monascus* spp. and *Aspergillus niger* inspired from Black-Skin-Red-Koji by a double-sided petri dish. *Front. Microbiol.* 12:670684. 10.3389/fmicb.2021.670684 34177849 PMC8221429

[B50] Zhang Y. R. Chen Z. T. Wen Q. Y. Xiong Z. X. Huang Z. W. (2020). An overview on the biosynthesis and metabolic regulation of monacolin K/lovastatin. *Food Funct.* 11 5738–5748. 10.1039/D0FO00691B 32555902

[B51] Zhang Y. Y. Zhu X. Y. Li X. Z. Tao Y. Jia J. He X. (2017). The process-related dynamics of microbial community during a simulated fermentation of Chinese strong-flavored liquor. *BMC Microbiol.* 17:196. 10.1186/s12866-017-1106-3 28915790 PMC5603089

[B52] Zheng X. W. Han B. Z. (2016). Baijiu, Chinese liquor, history, classification and manufacture. *J. Ethn. Foods* 3 19–25. 10.1016/j.jef.2016.03.001

[B53] Zhou K. X. Wu L. Chen G. M. Liu Z. B. Zhao X. Z. Lv X. (2021). Development of a novel restrictive medium for *Monascus* enrichment from Hongqu based on the synergistic stress of lactic acid and ethanol. *Front. Microbiol.* 12:702951. 10.3389/fmicb.2021.702951 34234769 PMC8256164

[B54] Zhu D. Jiang Y. L. Gao H. Huang Y. X. Wang H. (2014). Isolation and screening of red pigment-producing strains from Jiangxiang red-core Daqu. *Liq. Making Sci. Technol.* 10 27–31. 10.13746/j.njkj.2014.0217

