# Supplementary material for: Study on microbial community of “green-covering” Tuqu and the effect of fortified autochthonous Monascus purpureus on the flavor components of light-aroma-type Baijiu
Source: Front Microbiol. 2022 Aug 18;13:973616. doi: 10.3389/fmicb.2022.973616 (PMC9434108; doi:10.3389/fmicb.2022.973616)
Supplement: Supplementary file 1 [file Data_Sheet_1.docx]

Study on microbial community of "green-covering" *Tuqu* and the effect of fortified autochthonous *Monascus purpureus* on the flavor components of light-aroma-type *Baijiu*

Liping Zhu^1^, Lanqi Li^1^, Qiang Yang^1^, Liang Chen^1^, Lei Zhang^1^, Gang Zhang^1^, Bin Lin^1^, Jie Tang^1^, Zongjie Zhang^2^, Shenxi Chen^1*^

^1^*Hubei Key Laboratory of Quality and Safety of Traditional Chinese Medicine* *& Health Food, Jing Brand Co., Ltd,* *Daye, China*

^2^*Hubei Key Laboratory of Edible Wild Plants Conservation & Utilization, College of Life Sciences,* *Hubei Normal University, Huangshi, China*

**Contents** **Page**

1. Figure S1. The fermentation temperature and moisture curves of the production process of TQ and TQ’s brewing process 3
2. Figure S2. Appearance characteristics of experimental "red heart" *Tuqu* and NRH1 4
3. Table S1. Reads, OTUs, and alpha-diversity indexes 5
4. Table S2. The results of enzyme activity of *Monascus* 6
5. Table S3. The maximum tolerance of *Monascus* to ethanol concentration, temperature, and pH 7
6. Table S4. The volatile components contents and ethanol yield in original liquor fermented by NRH1 and ERH 8

Figure S1. The fermentation temperature and moisture curves of the production process of TQ and TQ’s brewing process. (A) The fermentation temperature curve of the production process of TQ. (B) The fermentation moisture curve of the production process of TQ. (C) The saccharification temperature curve of TQ’s brewing process. (D) The fermentation temperature curve of TQ’s brewing process.


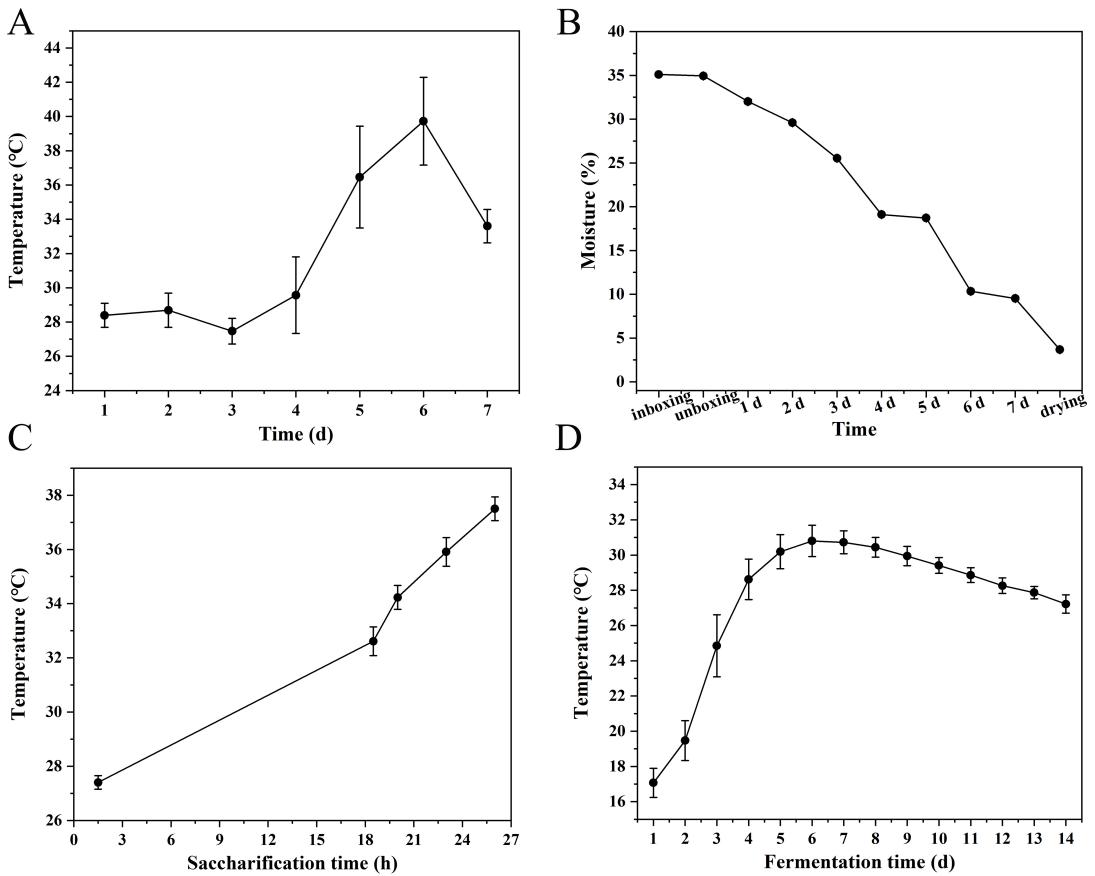


Figure S2. Appearance characteristics of experimental "red heart" *Tuqu* and NRH1.


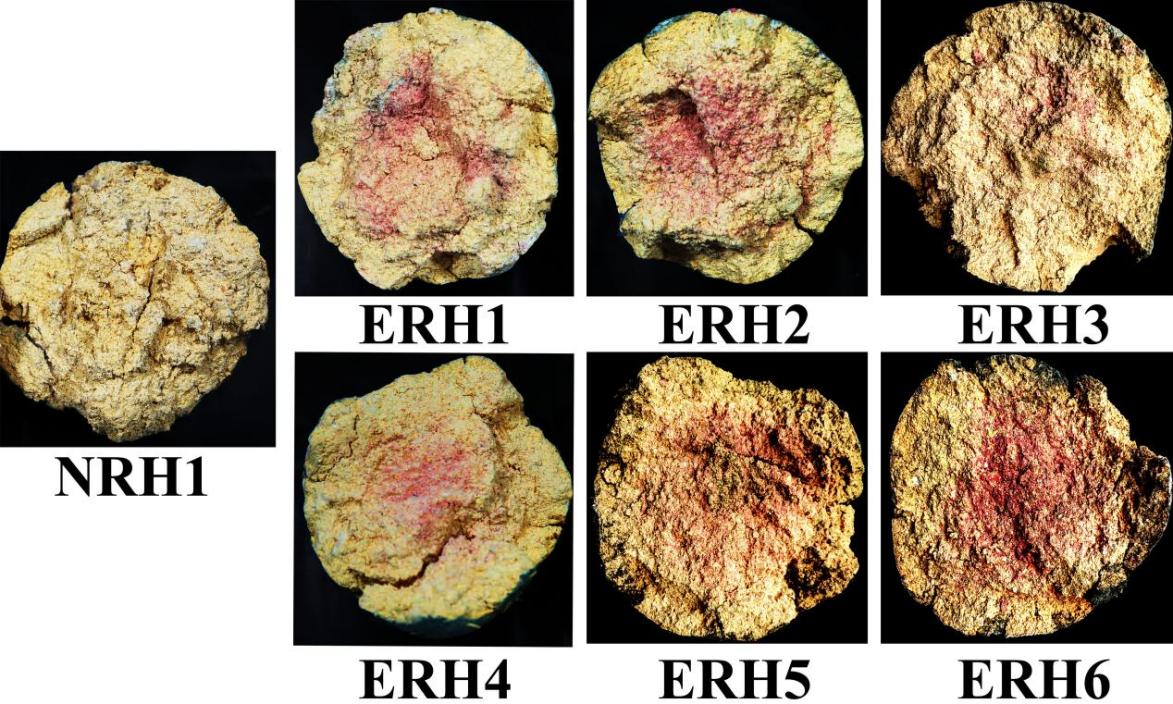


Table S1. Reads, OTUs, and alpha-diversity indexes.

| Samples | Reads | OTUs | ACE | Chao1 | Shannon | Simpson |
| --- | --- | --- | --- | --- | --- | --- |
| NRH | 155497 | 148 | 227.07 | 176.97 | 2.71 | 0.79 |
| RH | 155232 | 142 | 262.07 | 325.11 | 1.66 | 0.48 |

Table S2. The results of enzyme activity of *Monascus.*

| Samples | Protease activity (U/g) | Saccharification enzyme activity (U/g·h) | Esterase activity (mg/g·100 h) |
| --- | --- | --- | --- |
| M1 | 78.89 | 9533.46 | 5.18 |
| M2 | 30.14 | 5132.2 | 4.05 |

Table S3. The maximum tolerance of *Monascus* to ethanol concentration, temperature, and pH.

| Samples | Ethanol (%) | Temperature (℃) | pH |
| --- | --- | --- | --- |
| M1 | 23 | 45 | 3 |
| M2 | 21 | 45 | 3 |

Table S4. The flavor compounds contents and ethanol yield in original liquor fermented by NRH1 and ERH.

| Flavor compounds (mg/L) | NRH1 | ERH1 | ERH2 | ERH4 |
| --- | --- | --- | --- | --- |
| Acetaldehyde | 189.14 ± 123.51 | 41.39 ± 4.19 | 40.26 ± 1.75 | 494.02 ± 357.12 |
| Acetal | 72.94 ± 26.84ab | 20.07 ± 1.92b | 18.63 ± 1.82b | 273.42 ± 182.03a |
| Methanol | 48.02 ± 1.71 | 48.43 ± 3.68 | 43.91 ± 1.76 | 45.47 ± 3.23 |
| 2-butanol | 0.92 ± 0.07b | 1.32 ± 0.08a | 1.07 ± 0.00ab | 0.92 ± 0.13b |
| Propanol | 446.24 ± 10.94 | 441.86 ± 74.93 | 383.62 ± 4.92 | 411.82 ± 26.94 |
| Isobutanol | 521.59 ± 2.14a | 419.79 ± 26.81bc | 379.58 ± 7.02c | 450.00 ± 27.03b |
| Butanol | 4.54 ± 0.13 | 7.23 ± 1.99 | 5.65 ± 0.21 | 8.07 ± 0.55 |
| Isoamyl alcohol | 775.61 ± 1.16a | 562.83 ± 39.53bc | 491.96 ± 8.73c | 607.63 ± 39.47b |
| Pentanol | 1.09 ± 0.04 | 1.31 ± 0.18 | 1.36 ± 0.06 | 1.17 ± 0.23 |
| Hexanol | 1.12 ± 0.01b | 1.58 ± 0.13a | 1.47 ± 0.12ab | 1.42 ± 0.23ab |
| β-phenethyl alcohol | 12.83 ± 0.22 | 10.81 ± 0.59 | 11.75 ± 0.96 | 11.46 ± 0.22 |
| Ethyl formate | 11.56 ± 1.95 | 14.12 ± 4.63 | 12.71 ± 0.82 | 13.38 ± 4.43 |
| Ethyl acetate | 1295.60 ± 24.24c | 2604.33 ± 26.41b | 2901.29 ± 34.26a | 2540.83 ± 97.89b |
| Ethyl butyrate | 4.45 ± 0.04 | 6.03 ± 1.83 | 3.46 ± 0.12 | 3.70 ± 1.16 |
| Ethy hexanoate | 1.34 ± 0.00 | 0.86 ± 0.04 | 0.74 ± 0.12 | 1.29 ± 0.46 |
| Ethyl lactate | 168.74 ± 2.07b | 377.09 ± 102.35ab | 442.74 ± 49.74a | 230.95 ± 33.49ab |
| Ethyl caprylate | 1.70 ± 0.16b | 2.25 ± 0.13a | 1.66 ± 0.10bc | 1.30 ± 0.08c |
| Ethyl caprate | 0.85 ± 0.00 | 0.80 ± 0.07 | 0.78 ± 0.16 | 0.71 ± 0.10 |
| Isoamyl acetate | 7.12 ± 0.19c | 9.00 ± 0.19b | 9.77 ± 0.17a | 9.47 ± 0.31ab |
| Ethyl hexadecylate | 43.78 ± 5.60 | 64.34 ± 10.89 | 66.50 ± 10.89 | 59.45 ± 8.58 |
| Acetic acid | 262.93 ± 0.89c | 475.55 ± 23.29b | 641.96 ± 54.32a | 436.56 ± 68.31b |
| Propionic acid | 2.49 ± 0.23b | 3.64 ± 0.31ab | 4.13 ± 0.71a | 2.56 ± 0.57ab |
| Isobutyric acid | 11.43 ± 2.22ab | 13.49 ± 0.82a | 11.59 ± 1.00ab | 9.01 ± 0.21b |
| Isovalericacid | 1.49 ± 0.24b | 2.45 ± 0.33a | 2.06 ± 0.18ab | 1.59 ± 0.25b |
| 3-hydroxy-2-butanone | 3.86 ± 3.19 | 3.27 ± 2.99 | 1.98 ± 0.14 | 18.98 ± 21.41 |
| Alcohol yield | 45.38 ± 0.36 | 44.65 ± 1.12 | 42.57 ± 1.27 | 44.19 ± 0.36 |

* Ethanol yield represents the weight of 55% volume fraction of ethanol divided by the weight of grain.

Sample groups with different letters (a, b,c) mean significant differences (*p* < 0.05) as determined by one-way ANOVA Turkey’ HSD test.
